# Analysis of the Binding of Analyte-Receptor in a Micro-Fluidic Channel for a Biosensor Based on Brownian Motion

**DOI:** 10.3390/mi11060570

**Published:** 2020-06-03

**Authors:** Sunghak Choi, Woo Il Lee, Gyu Hee Lee, Yeong-Eun Yoo

**Affiliations:** 1 School of Mechanical and Aerospace Engineering, Seoul National University, 1 Gwanak-ro, Gwanak-gu, Seoul 08826, Korea; chsh1123@gmail.com (S.C.); wilee@snu.ac.kr (W.I.L.); gyuool@snu.ac.kr (G.H.L.); 2 Department of Nano Manufacturing Technology, Korea Institute of Machinery and Materials, 156 Gajeongbuk-Ro, Yusung-Gu, Daejeon 34103, Korea

**Keywords:** biosensor, specific binding of analyte-receptor, transport model for micro-fluidic channel based on Brownian motion

## Abstract

This study experimentally analyses the binding characteristics of analytes mixed in liquid samples flowing along a micro-channel to the receptor fixed on the wall of the micro-channel to provide design tools and data for a microfluidic-based biosensor. The binding or detection characteristics are analyzed experimentally by counting the number of analytes bound to the receptor, with sample analyte concentration, sample flow rate, and the position of the receptor along the micro-channel length as the main variables. A mathematical model is also proposed to predict the number of analytes transported and bound to the receptor based on a probability density function for Brownian motion. The coefficient in the mathematical model is obtained by using a dimensionless mathematical model and the experimental results. The coefficient remains valid for all different conditions of the sample analyte concentration, flow rate, and the position of the receptor, which implies the possibility of deriving a generalized model. Based on the mathematical model derived from mathematical and experimental analysis on the detection characteristics of the microfluidic-based biosensor depending on previously mentioned variables and the height of the micro-channel, this study suggests a design for a microfluidic-based biosensor by predicting the binding efficiency according to the channel height. The results show the binding efficiency increases as the flow rate decreases and as the receptor is placed closer to the sample-injecting inlet, but is unaffected by sample concentration.

## 1. Introduction

**Figure 1:**
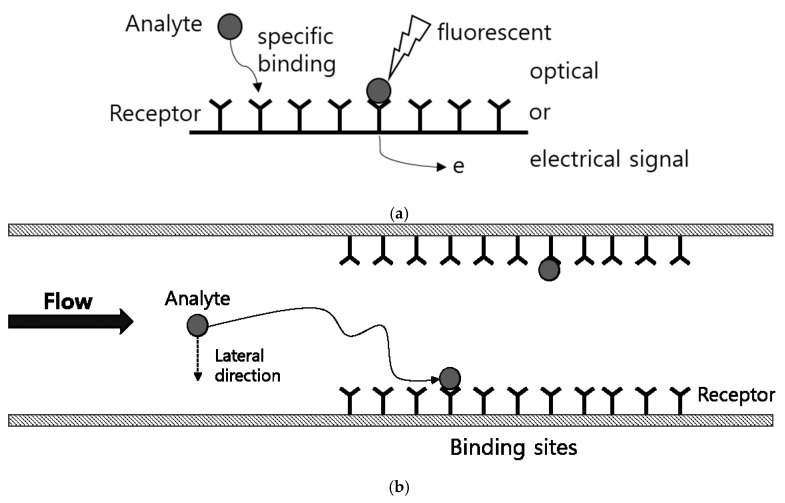
(**a**) Schematic of sensing mechanism for bio sensor. (**b**) Schematic of binding process between analyte and receptor in micro-channel.

Biosensors are devices used to detect specific analytes based on antigen-antibody reactions [1]. Detection using biosensors is achieved through a process in which measurable optical or electrical signals are derived from the binding of an analyte and a receptor specific to this analyte (Figure 1a) [2,3,4,5]. Biosensors are used in various fields, including medical diagnostics, which require highly sensitive detecting of low-concentration analytes below ng/mL for early diagnosis or precise analysis [1,6,7,8]. To achieve this, a wide variety of studies are being conducted on improving the binding efficiency between analytes and receptors, or the signal efficiency and intensity from the analyte-receptor binding [9,10,11,12,13]. In terms of binding efficiency, it is important to bring the analyte within a distance where binding is possible in addition to improving the binding affinity between receptors and analytes. Adapting micro/nano-channels or beads for higher surface-to-volume ratio is an effective method to increase the binding probability between low concentration of analytes in liquid samples and receptors fixed on the surface of the channels or beads such as micro-fluidic biosensor [1,14,15,16,17].

In the case of microfluidic-based biosensors, despite adapting micro-channel for higher chance of analyte-receptor binding, it may be hard to place the analytes close enough, for example within 20 nm [18], to the receptors while flowing through the micro-channel, considering the channel size, analyte size, and the strong laminar flow in the micro-channel. The probability decreases for lower concentration of analytes mixed in the sample, which results in poor detection ability of biosensors. Therefore, in addition to research on improving analyte-receptor binding kinetics or the characteristics of optical or electrical signals derived from bound analyte-receptor, quantitative analysis on the behavior of analytes flowing along a micro-channel is required to improve the detection characteristics of microfluidic-based biosensors. 

As mentioned above, in the case of microfluidic-based biosensors with receptors fixed to the wall of the micro-channel, the binding between the analyte and receptor occurs on the wall of the channel as shown in Figure 1b. Therefore, the analytes should move toward the micro-channel wall during the flow for sensing. However, it is usually difficult to generate a lateral flow to the wall. Various studies have been performed to enhance transportation of analytes towards the wall by external fields [19,20,21] or vortices caused by mixing structures on the surface [22,23,24], but using external energy or fabricating additional surface structures may not always be practical due to cost, user environments or manufacturing issues. When no external field or mixing surface structure is present for lateral flow, the movement of analytes such as enzymes, proteins, viruses, or bacteria is mainly driven by Brownian motion [25,26,27]. Therefore, analyzing the binding properties between the analyte and receptor by considering the Brownian motion of the analyte is critical for developing highly sensitive microfluidic biosensors.

Transport of analytes inside microfluidic channel is usually described by the reaction-diffusion model and it is widely accepted. Numerical simulation for a rectangular channel with micro-pillar was performed [28] and diffusion-convection model for flat plates was described considering the standard entrance effect [29]. Moreover, the transport of analytes and surface reaction was numerically modeled for the electro-osmotic flow [30]. However, it is difficult to reflect the individual motion of analyte such as shear induced lift force movement because it is described in the perspective of concentration, which is regarded as important effect in a micro-sized channel. Thus, a transport model needs to be presented in the individual analyte perspective, especially for the low-concentration case in which continuum hypothesis may not be valid. This study suggests a model for the transport of analytes to receptors fixed on the micro-channel in a microfluidic-based biosensor using a mathematical model by calculating the number of analytes capable of colliding on the wall based on Brownian motion. This study also experimentally examines the number of bound particles according to the design parameters of the device, such as the size of the channel or the position of the receptor, and the parameters for the diagnostic process, such as the concentration and flow rate of the sample. The findings are compared with the results calculated by the model, and a design for the main structure of the microfluidic-based biosensor is presented using the mathematical model.

## 2. Experiments

**Figure 2:**
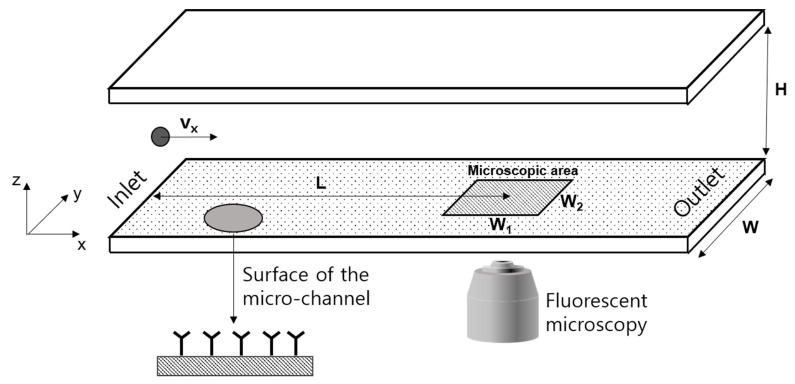
Configuration of the micro-fluidic biosensor for analyte-receptor binding experiments.

To examine the analyte-receptor binding efficiency depending on the detection conditions such as the concentration of the sample, flow rate, and the receptor-analyte binding measurement position, this study counted the number of bound particles while the sample including analytes flowed through a microchannel (height: 100 µm) as shown in Figure 2. In terms of the model receptor, streptavidin was immobilized on the micro-channel surface. As for the analyte, this study used fluorescent biotinylated Polystyrene beads (FluoSpheres™ Biotin-Labeled Microspheres, diameter: 200 nm) with excellent binding properties with streptavidin [31,32]. The binding between streptavidin-biotin was confirmed by measuring the fluorescence of fluorescent polystyrene beads. Also, by using engineering particles uniform in size and density as a model analyte, the number of analytes used in the experiment was able to be estimated for each condition, which enabled more accurate measurement and analysis of receptor-analyte binding properties according to the concentration. The next section describes the experimental method and conditions including surface treatment for streptavidin immobilization.

### 2.1. Microfluidic Channel and Surface Modification

A micro-fluidic channel was designed to be 100 µm in depth (*H* = 100 µm) and 5 mm in width (*W* = 5 mm) to reduce the effect of the side wall of the channel on the flow. The length of the channel was set to 70 mm. A transparent micro-fluidic channel substrate was injection molded using PMMA (Poly methyl methacrylate) to observe the behavior of the particles and fluorescent image of the Polystyrene (PS) particles bound to the streptavidin. 

For biotinylated beads-streptavidin binding, streptavidin is required to be coated previously on the PMMA micro-channel wall, which consists of several processes as follows. After sonication in iso-propanol (IPA) for 15 min, PMMA substrates were washed with IPA and DI water in turn [33]. The surface of the micro-channel was carboxylated by exposing to UV light for 30 min for effective streptavidin coating. Streptavidin was immobilized onto the PMMA surface by dipping in coating solution, a mixture of 50 µL of streptavidin, 25 mg of EDC (1-ethyl-3-(3-dimethylaminopropyl) carbodiimide) reagent, and 5 mL of MES buffer. EDC was used for facilitating binding of the amino part of streptavidin with carboxyl groups on the PMMA surface. After incubation for 2 h 30 min, the PMMA substrates were washed and dried with nitrogen gas [34].

### 2.2. Investigation for Bound Particles

The experiment was performed by flowing the samples during each detection time. After injecting the solution containing beads, the microchannel was washed three times by 10 mL of deionized water for each washing. DI water was injected at flow rate 10 mL/h in order to exclude non-intended adsorption of beads. Non-specific binding of biotinylated PS beads was investigated through bare PMMA surface. Because the experiments were performed under flow and the bead solution used for the experiments contained Tween20, none of the particles were bound to bare PMMA after washing. 

**Figure 3:**
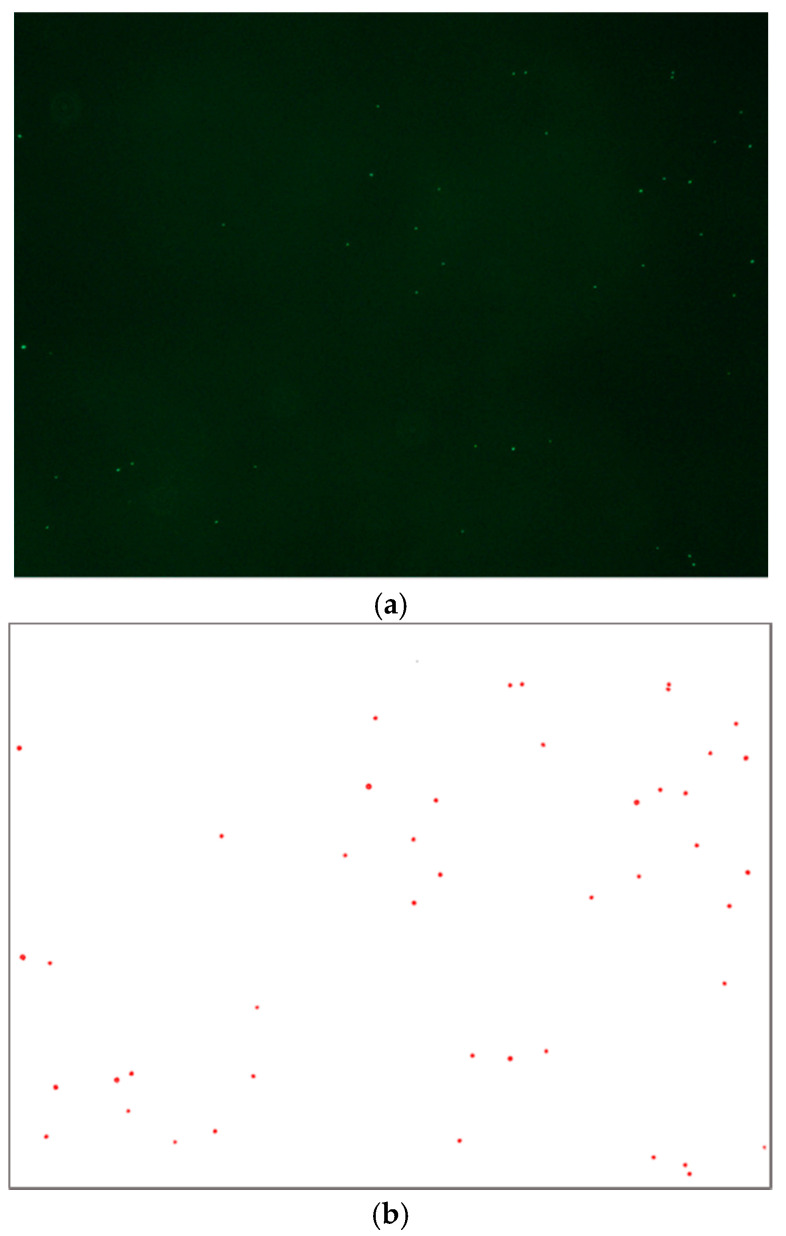
Image for fluorescent biotinylated Polystyrene (PS) bead bound to streptavidin on the surface of the micro-channel (**a**) from fluorescent microscopy (**b**) after image processing for counting the bound beads.

Images of the particles bound to a specific area (*W*_1_ = 1362 µm, *W*_2_ = 1021 µm) of the channel wall were obtained with a fluorescence microscope. As shown in Figure 3, after setting a binary threshold to remove the noise and image processing for the uniform size of the fluorescence image of the particle, the number of particles bound in the microscopic area shown in Figure 2 were counted discretely using distributed fluorescent signals. Every experiment used different channel plate with same micro-channel in dimension, material and surface conditions.

### 2.3. Experimental Conditions

The experiments were repeated five times each per the conditions shown in Table 1 below. The flow rate conditions were 0.3 mL·h^−1^, 1.2 mL·h^−1^, and 2.4 mL·h^−1^. In the case of 1.2 mL·h^−1^, the average velocity of the flow ($\bar{v_{x}}$) is equivalent to about 0.67 mm·s^−1^. In addition, the bound particles were observed at different observation positions from the inlet to 10 mm, 35 mm, and 60 mm, respectively, to investigate the effect according to the sample flow distance in the micro-channel.

## 3. Mathematical Model and Analysis

**Figure 4:**
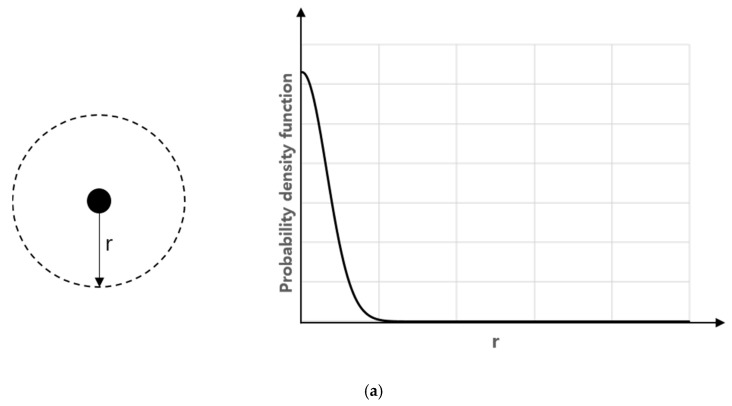

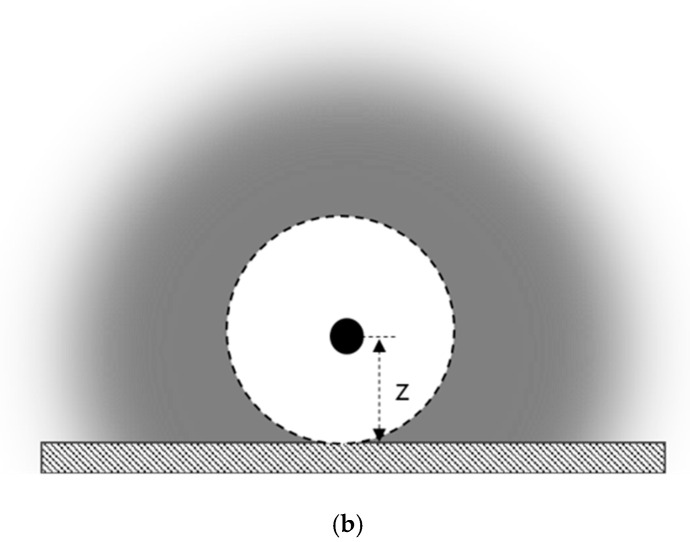
(**a**) Probability density function of particles existing at distance *r* from the initial position after *t*. (**b**) Schematic diagram of the probability that a particle at a distance of *z* from the channel wall will reach the wall after *t*.

**Figure 5:**
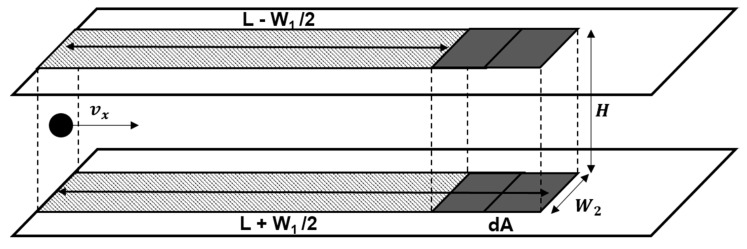
Geometrical configuration of mathematical model for analyte-receptor binding on microscopic area of a model description.

As shown in Figure 4a, the probability density function that a particle may exist at a distance (*r*) from the initial position after a certain time (*t*) is shown in Equation (1) by Einstein’s Brownian motion [26]. Therefore, as shown in Figure 4b, the probability *P*(*z*) of a particle at a distance (*z*) from the channel wall to collide with the wall by Brownian motion during a certain time (*t*) can be expressed as an integral form of Equation (1) for positions farther than *z*, as shown in Equation (3). A correction factor *C*_1_ is introduced considering a microscopic area limited only to a specific direction towards wall (Figure 5).

(1) $$p\left(r\right)=\frac{1}{\sqrt{4\pi Dt}}e^{\frac{-r^{2}}{4Dt}}$$

where *D* is the diffusion coefficient ($m^{2}\cdot s^{-1}$), *t* is the elapsed time (s), *r* is the distance from the initial position of the particle (m).

Brownian motion is affected by the temperature, the viscosity, and the radius of analyte. In this study, these properties are fixed so that the diffusion coefficient is constant. The diffusion coefficient *D* is calculated (*D* = $2.4\times 10^{-12}{\;m}^{2}\cdot s^{-1})$ from the Stokes-Einstein relation as shown in Equation (2):

(2) $$D=\frac{k_{B}T}{6\pi \eta r}$$

where $k_{B}$ is the Boltzmann constant, *T* is the absolute temperature, η is the viscosity, *r* is the radius of analyte.

(3) $$P\left(z\right)=C_{1}\int_{z}^{\infty }dr\frac{1}{\sqrt{4\pi Dt}}e^{\frac{-r^{2}}{4Dt}}$$

If the density function for the spatial distribution of particles is *f*(*x*,*y*,*z*), the total number of particles colliding with the wall during *t* is as below.

(4) $$N_{H,\mathrm{total}}=C_{1}\int \int \int f\left(x,y,z\right)P\left(z\right)\;dV$$

The density function may be considered as a constant assuming that the particles are distributed uniformly in the channel, and expressed as *f*(*x*,*y*,*z*) = *n* by using number density *n*.

Since not all the particles that collide with the wall are bound to the receptor, the number of particles bound to the microscopic area can be expressed as follows by introducing coefficient *C*_2_ to reflect the collide-to-bound ratio and integrating it with *C*_1_ above into coefficient *C* = *C*_1_*C*_2_.

(5) $$N_{\mathrm{bound}}=C_{2}N_{H,\mathrm{total}}$$

If there is a flow, the total amount of particles involved during the detection time (*τ*) is calculated by using the number of particles that passed through the channel cross-section. When the *x*-direction velocity of the particle is *v_x_*, as shown in Figure 5, the volume of the space where the injected particles are distributed is $W_{2}\int_{0}^{H}v_{x}\left(z\right)\tau dz$. Therefore, the total number of injected particles can be expressed using number density *n* as follows.

(6) $$nW_{2}\int_{0}^{H}v_{x}\left(z\right)\tau dz$$

In addition, *t* varies depending on the distance (*L*) from the inlet. As shown in Figure 5, *t* during the transport of particles from the inlet to points *L* − *W*_1_/2 and *L* + *W*_2_/2 by the flow are $\frac{L-W_{1}/2}{v_{x}}\;\mathrm{and}$ $\frac{L+W_{2}/2}{v_{x}}$, respectively. Therefore, the number of particles bound to the microscopic area (dA) with a distance of *L* from the inlet can be expressed as the difference between when *t* is $\frac{L+W_{1}/2}{v_{x}}$ and $\frac{L-W_{1}/2}{v_{x}}$ as shown below.

(7) $$N_{\mathrm{bound}}=CnW_{2}\left[\int_{0}^{H}{P\left(z\right)|}_{t=\frac{L+W_{1}/2}{v_{x}}}\;v_{x}\left(z\right)\tau \;dz-\int_{0}^{H}{P\left(z\right)|}_{t=\frac{L-W_{1}/2}{v_{x}}}\;v_{x}\left(z\right)\tau \;dz\right]$$

where *n* is number density (m^−3^), *L* is the distance from inlet of microscopic area (m), *H* is the height of channel (m), *τ* is detection time (s), $v_{x}$ is the *x*-directional speed of flow (m·s^−1^), *W*_1_ and *W*_2_ are the length and width of microscopic area dA, respectively (m).

Equation (7) predicts the number of bound particles by using the displacement of individual particles being diffused independently by Brownian motion and the total number of injected particles, in which the Brownian motion is not affected by the flow. 

## 4. Results and Discussion

### 4.1. The Number of Bound Particles

**Figure 6:**
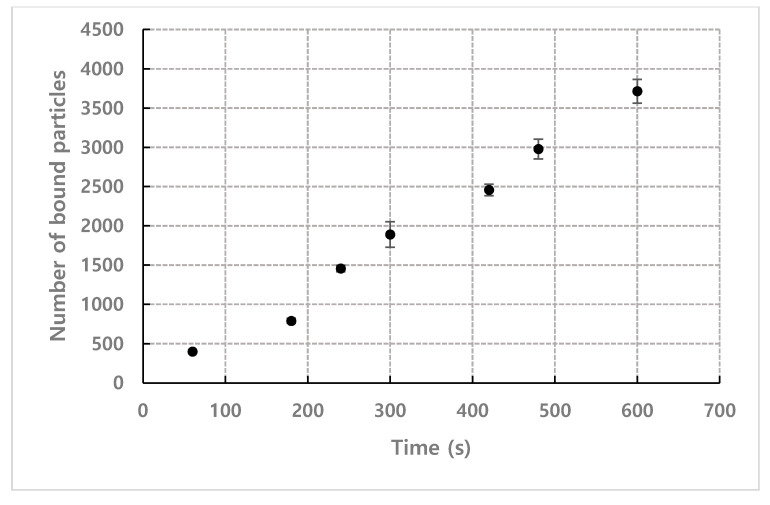
Result of measuring the number of bound particles according to the detection time (*n* = $1.93\times 10^{15}\;m^{-3}$, flow rate = 1.2 mL·h^−1^, observation position = 35 mm).

Figure 6 shows the results of measuring the number of bound particles according to the detection time under the conditions of number density *n* = $1.93\times 10^{15}\;m^{-3}$, flow rate = 1.2 mL·h^−1^, and observation position = 35 mm. The number of bound particles increased linearly over time, which is consistent with *N*_bound_ being linear to the detection time (*τ*) in Equation (7). 

**Figure 7:**
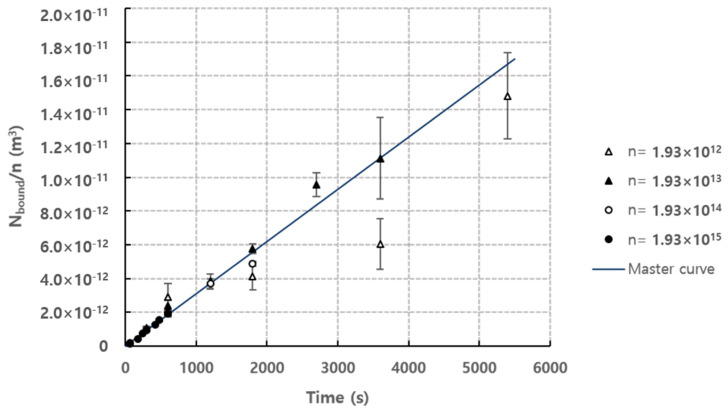
Normalized *N*_bound_ according to the time for each concentration (Normalized by number density *n*), flow rate = 1.2 mL·h^−1^, observation position = 35 mm. Master curve was fitted using the least square method ($R^{2}=0.90$).

Under the conditions of flow rate = 1.2 mL·h^−1^ and observation position = 35 mm, Figure 7 shows the results of the number of bound particles normalized by number density n according to the detection time for each concentration. As shown in the graph, the normalized values approximate to a single linear curve.

**Figure 8:**
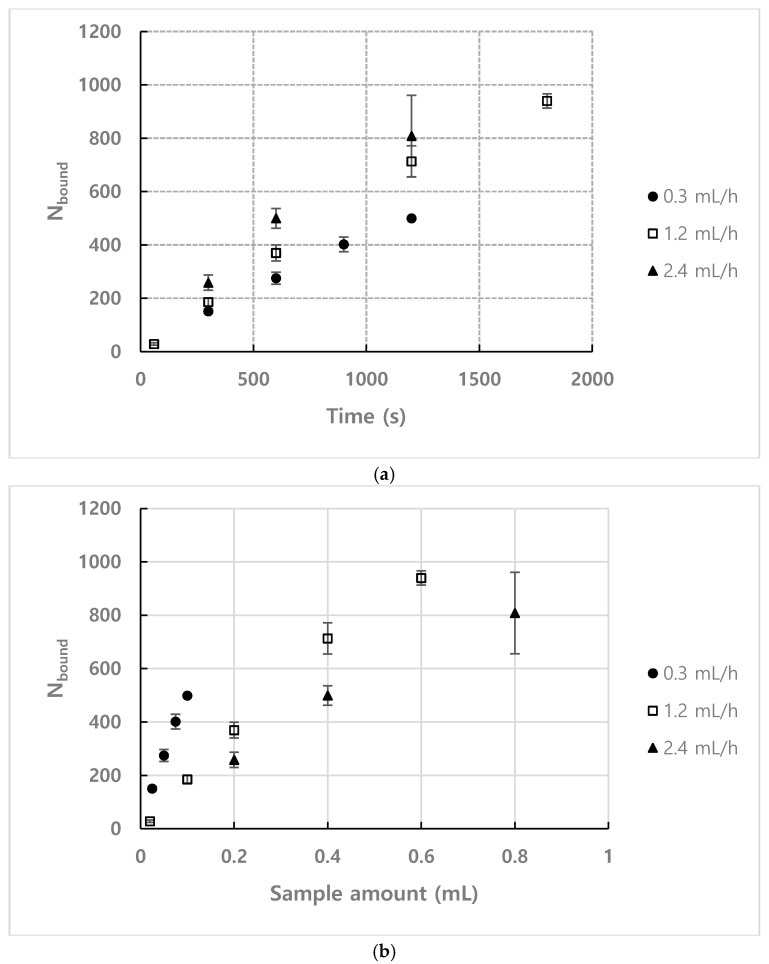
Number of bound particles depending on flow rate for (**a**) *n* = $1.93\times 10^{14}\;m^{-3}$, observation position = 35 mm (**b**) *n* = $1.93\times 10^{14}\;m^{-3}$, observation position = 35 mm.

Figure 8a shows the result of plotting the number of bound particles for each flow rate over time under the conditions of *n* = $1.93\times 10^{14}\;m^{-3}$ and observation position = 35 mm. Figure 8b shows the number of bound particles compared to the quantity of the sample injected to directly compare the binding efficiency. Since the number density of the sample used in the experiment was $1.93\times 10^{14}\;m^{-3}$, 0.1 mL of the sample contains approximately $1.93\times 10^{7}$ analytes. As shown in Figure 8b, the binding efficiency increases as the flow rate decreases. This is because the traveling time for particles to be bound to the microscopic area by Brownian motion increases for lower flow rate. Considering these results, lower flow rate is better to improve detection efficiency when using the same quantity of samples.

**Figure 9:**
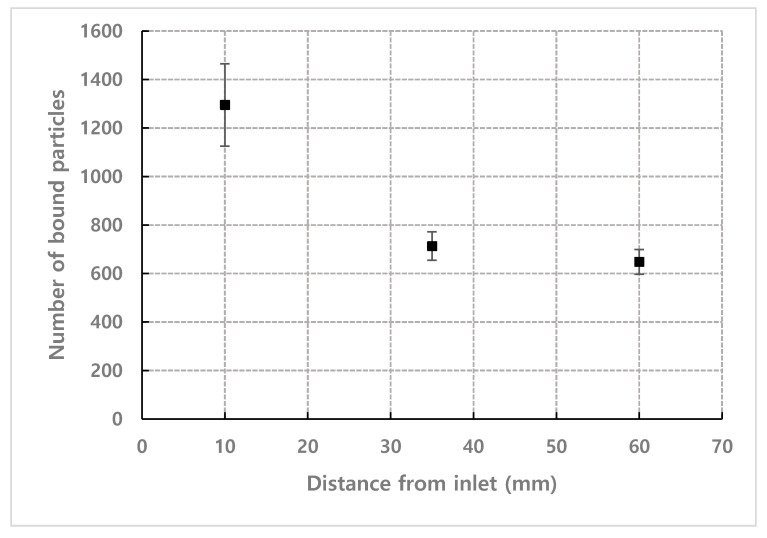
Number of bound particles depending on the distance of the observation position from inlet (*n* = $1.93\times 10^{14}\;m^{-3}$, τ = 1200 s, Flow late = 1.2 mL·h^−1^).

Figure 9 shows the result of counting the number of bound particles at the different observation positions under the conditions of *n* = $1.93\times 10^{14}\;m^{-3}$, τ = 1200 s and flow rate = 1.2 mL·h^−1^. The number of bound particles tended to decrease exponentially as the distance from the inlet increased. Therefore, it is favorable to place the receptor in microfluidic-based biosensors as close as possible to the inlet where samples are injected.

### 4.2. Numerical Estimation for the Number of Bound Particles based on Mathematical Model

**Figure 10:**
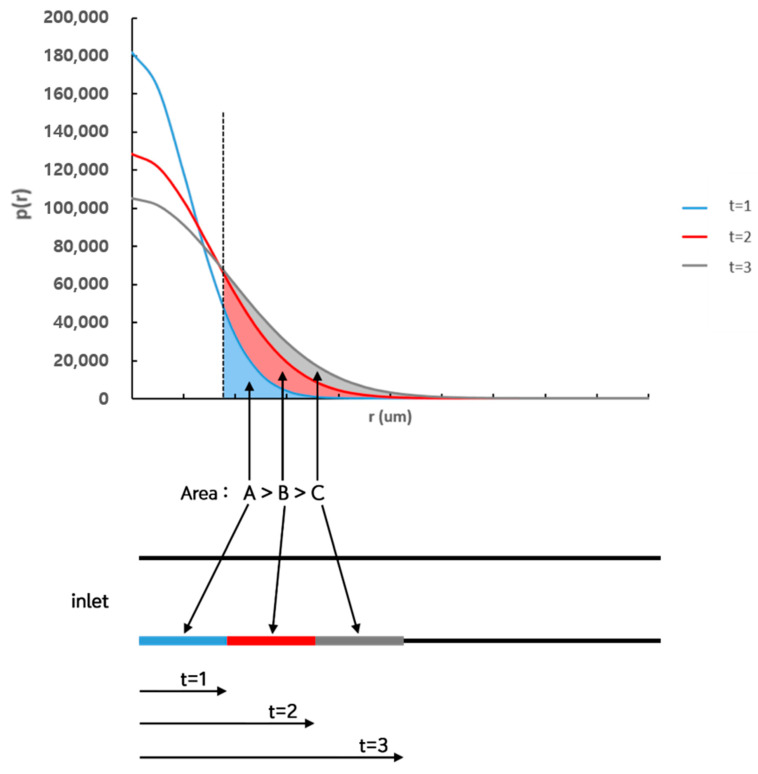
Increment of the probability of which a particle bound to the surface as time increases after being injected through inlet to the microscopic area further off from inlet.

The particles mixed with the liquid sample are injected into the inlet flow along the micro-channel and pass through the microscopic area if they do not be bound to the receptor after hitting the channel wall by Brownian motion during time (*t_L_*) to reach observation position (*L*). Therefore, *t* = *t_L_* in the probability density function Equation (1), which is the basic equation for calculating the number of particles bound to the channel surface from the inlet to the microscopic area. When limiting the area to count the bound particles to a microscopic area with a distance of *L* from the inlet, the number of bound particles can be calculated as follows. The value is calculated by the difference between the number of particles bound to the surface of the channel during time $t_{L+\frac{W_{1}}{2}}$ required to transport to the position where the microscopic area ends and the number of particles bound to the surface of the channel during time $t_{L-\frac{W_{1}}{2}}$ required to transport to the position where the microscopic area starts. In this calculation, the time required for the particles to pass through the microscopic area is ($\frac{W_{1}}{v_{x}})$, which is the time it takes for the particles to pass through *W*_1_, regardless of the position of the microscopic area (*L*). As the traveling time increases as the observation position (*L*) moves further away from the inlet, the probability density function (Equation (1)) for the position of the particles becomes broad as shown in the graph in Figure 10. Thus, the integral value of the probability density function, which is the probability a particle exists over a certain distance from the initial position of the particle increases and the number of particles bound to the channel surface from the inlet is thereby increased. However, although the integral value of the probability density function over a certain distance increases as *t* increases, the increase rate decreases. Therefore, as *t* increases, the integral value for the increment Δ*t* of the same time, that is, the number of bound particles decreases. Therefore, as the microscopic area moves further away from the inlet, the transport time (*t*) of the particles to the corresponding position increases, while the time required to pass through the microscopic area of the same size (Δ*t*) is the same, resulting in a decrease in the number of particles bound to the microscopic area. 

### 4.3. Empirical Coefficient of Mathematical Model for Bound Particles

The experimental results for the number of bound particles normalized by number density under various particle concentration conditions (Figure 7) was used to obtain the empirical coefficient *C* of the mathematical model to calculate the number of bound particles of Equation (5) or Equation (7). Therefore, Equation (7) can be rewritten as below.

(8) $$\frac{N_{\mathrm{bound}}/n}{\tau }=CW_{2}\left[\int_{0}^{H}{P\left(z\right)|}_{t=\frac{L+W_{1}/2}{v_{x}}}\;v_{x}\left(z\right)\;dz-\int_{0}^{H}{P\left(z\right)|}_{t=\frac{L-W_{1}/2}{v_{x}}}\;v_{x}\left(z\right)\;dz\right]$$

In Figure 7, the number of normalized bound particles shows results that change linearly with respect to *τ*, so the slope of the linear curve corresponds to $\frac{N_{\mathrm{bound}}/n}{\tau }$ of Equation (8), and the value of the empirical coefficient *C* can be obtained by using the slope of this curve. The slope of the curve was $3.09\times 10^{-15}$ using the least square method, and therefore *C* = 0.0395. This empirical coefficient *C* for mathematical model for behavior of the analytes is estimated based on experimental results as explained above. Comparing works in this paper, it can be seen that some previous researchers, however, studied the binding kinetics near the receptors [28,29,30]. As shown in Equation (9), the binding rate of the analyte can be estimated based on the association-dissociation coefficient, analyte concentration near the wall or receptor, density of the receptors on the surface.

(9) $$\frac{\partial C_{s}}{\partial t}=k_{\mathrm{on}}C_{\mathrm{wall}}\left(C_{s0}-C_{s}\right)-k_{\mathrm{off}}C_{s}$$

where $C_{s}$ is the density of the analytes bound to receptor on the surface of the micro-channel, $C_{wall}$ is the analyte concentration near wall, $C_{s0}$ is the density of the receptors on the micro-channel surface, $k_{\mathrm{on}}$ and $k_{\mathrm{off}}$ are the association and dissociation rate constants, respectively.

Equation (9) can be expressed as below with the assumptions that the streptavidin has high affinity for biotin and is immobilized with high surface density onto PMMA surface ($k_{\mathrm{on}}\gg k_{\mathrm{off}}$, $C_{s0}\gg C_{s}$).

(10) $$\frac{\partial C_{s}}{\partial t}=k_{\mathrm{on}}C_{\mathrm{wall}}C_{s0}$$

Here, $C_{\mathrm{wall}}$ corresponds to the number of analytes transported to the wall, $N_{H,\mathrm{total}}$ in Equation (5). Thus, $C_{2}$ corresponds to the $k_{\mathrm{on}}C_{s0}$, semantically, but direct comparison is difficult because the model in this study describes transport of analytes in individual analytes perspective and focuses on the low-concentration case in which the density of the receptors outweighs the analyte concentration. 

**Figure 11:**
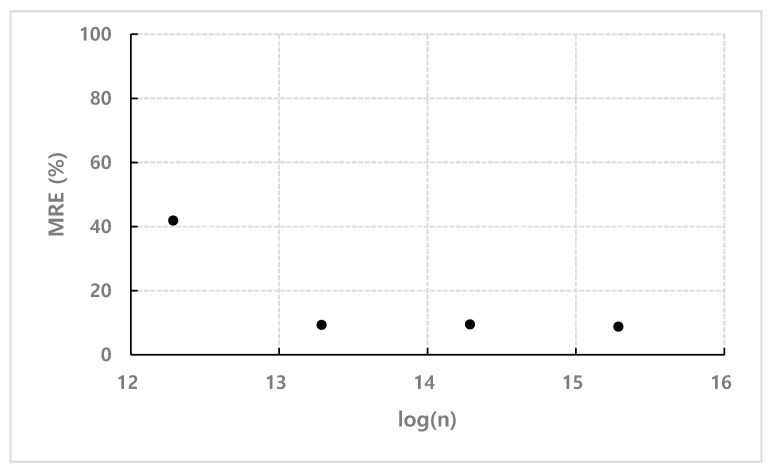
Mean relative errors (MRE) of experimental values according to the concentration and the values calculated by using the empirical coefficient *C*.

**Figure 12:**
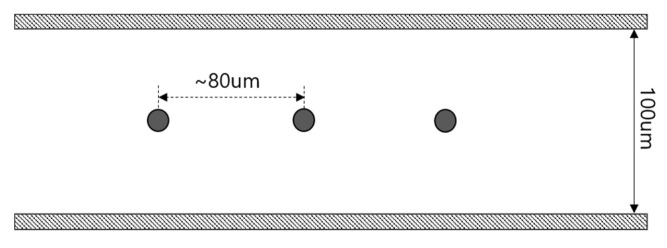
Interparticle distance when the concentration is *n* = $1.93\times 10^{12}\;m^{-3}$.

Figure 11 shows the mean relative errors (MRE) for each concentration to compare the results calculated by the empirical coefficient *C* with the experimental results. According to the graph, the values calculated by the mathematical model with the empirical coefficient *C* obtained from the normalized data in Figure 7 show a valid correlation with experimental values. However, there is a relatively large deviation in terms of number density *n* = $1.93\times 10^{12}\;m^{-3}.$ This is because of the difficulty in assuming the density function for the spatial distribution of particles as a constant in extremely low-concentration samples as in Section 3. In the case of number density *n* = $1.93\times 10^{12}\;m^{-3}$, considering that the interparticle distance is about 80 µm and the height of the channel used in the experiment is 100 µm, as shown in Figure 12, only one particle is distributed in the height direction on average. Consequently, it is difficult to apply a continuum mathematical model and a large deviation is shown. 

**Figure 13:**
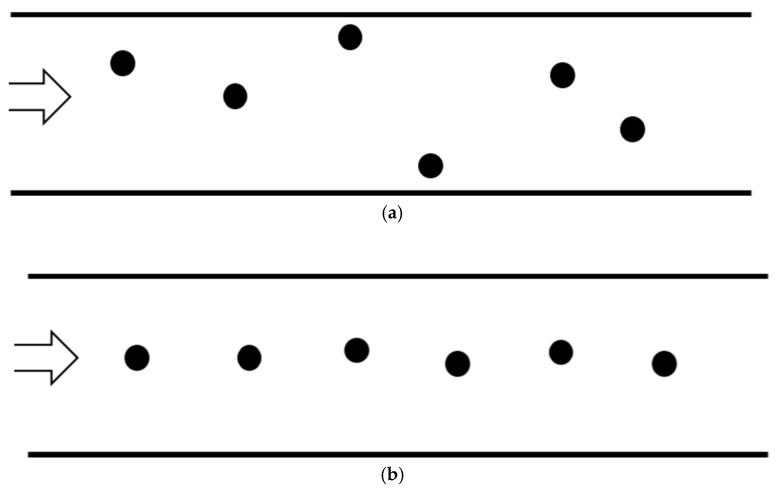
(**a**) Random dispersed particle. (**b**) Focused on center of channel due to the interaction between particle and wall.

The results also show that the interaction between particles and wall has almost no effect on the distribution of particles in the channel. This is because if the repulsion of the wall and particles affects the distribution in the channel, the particles will be concentrated near the center of the channel as shown in Figure 13b, which will reduce the binding efficiency significantly. That is, the wall-particle repulsion may act strongly near the wall, but not enough to affect the distribution of particles.

Meanwhile, the shear induced lift force can affect transport of analytes and it is described as below [35].

(11) $$F_{L}=K\mu Vr^{2}{\left(\frac{\dot{\gamma }}{\nu }\right)}^{1/2}$$

where *K* is the numerical constant, V is the relative velocity, *r* is the radius of particle, $\dot{\gamma }$ is the shear rate, μ and ν are the dynamic and kinetic viscosity, respectively.

The maximum lift force exerted to the analyte is $6.31\times 10^{-18}\;N$ from Equation (11) and Goldman results for the slip velocity [36]. For this maximum lift force, the maximum terminal velocity and time to reach terminal velocity are calculated as below.

(12) $$v_{t,\max}=\frac{F_{\mathrm{lift}}}{6\pi \mu r}\;\sim \;3.35\;\mathrm{nm}/s$$

(13) $$T=\frac{m}{6\pi \mu r}\;\sim \;2\times 10^{-12}\;s$$

Considering that it is the maximum value and it is dissipated as the analyte moves to the channel center, the effect by the shear can be neglected.

**Figure 14:**
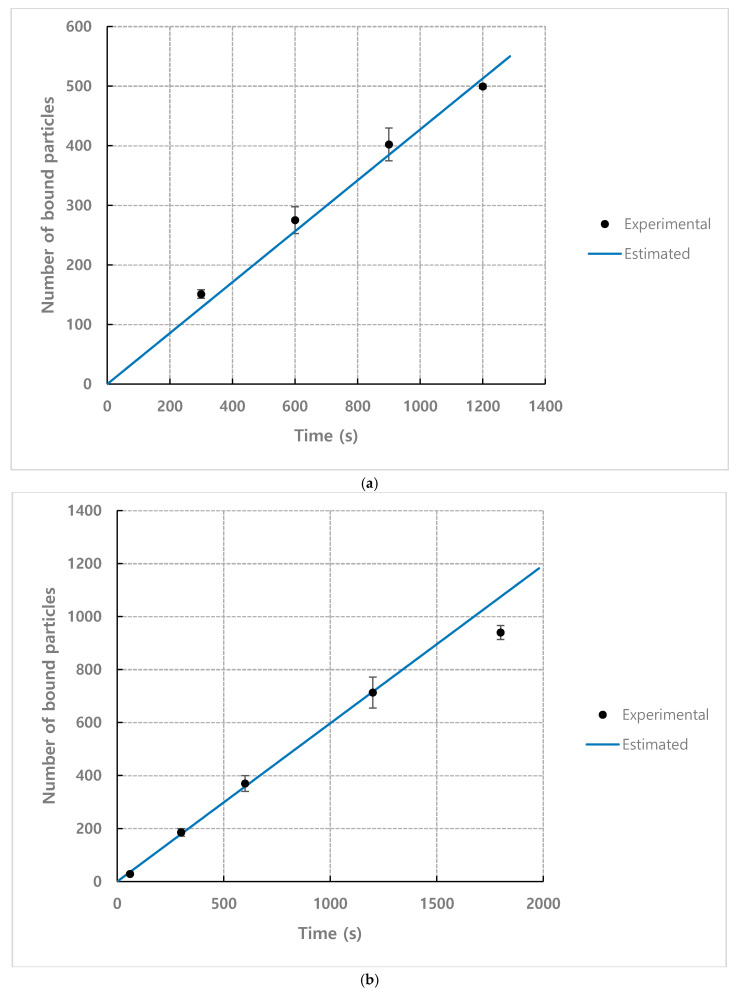

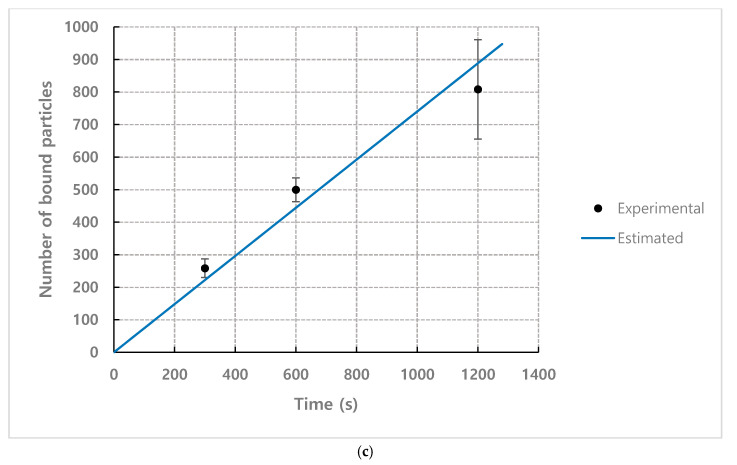
Comparison for number of bound particles from experiment and mathematical model (Equation (7)) depending on time for different flow rate condition (**a**) flow rate = 0.3 mL·h^−1^, (**b**) flow rate = 1.2 mL·h^−1^, (**c**) flow rate = 2.4 mL·h^−1^ (*n* = $1.93\times 10^{14}\;m^{-3}$, observation position = 35 mm). Solid lines depict data fitting with Equation (7). Coefficients of determination are (**a**) $R^{2}=0.98$, (**b**) $R^{2}=0.98$, (**c**) $R^{2}=0.93$.

The results of comparing the test results with different flow rates using coefficient *C* = 0.0395 with the predicted values by the model are as follows (Figure 14). Even when the flow rate conditions were different, the results predicted by the model were close to the experimental values. 

For capillary flow, which is widely used in medical diagnostic device, the flow rate is determined by the surface tension and the viscosity of sample solution. Except for the flow front, velocity profile for the capillary flow is coincident to the pressure driven flow. Thus, the mathematical model can be applied for capillary flow in the same manner with adequate velocity profile. 

### 4.4. Effect of Observation Position from Inlet and Channel Height

**Figure 15:**
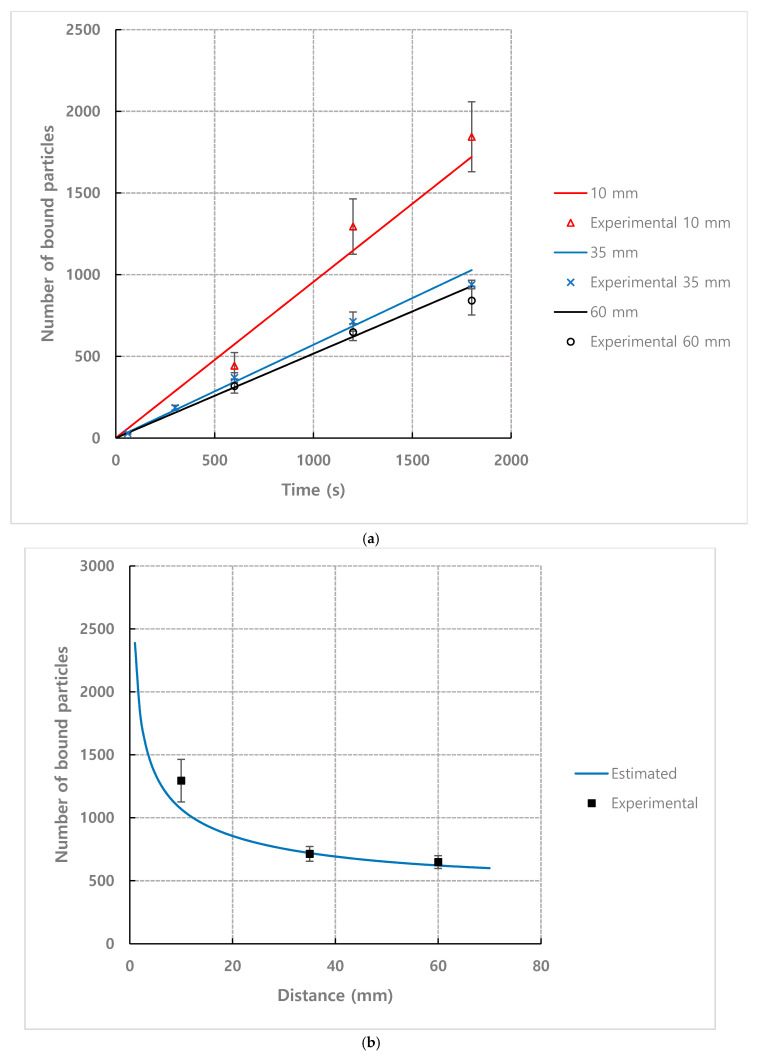
Comparison for number of bound particles from experiment and mathematical model (Equation (7)) for *n* = $1.93\times 10^{14}\;m^{-3}$, flow rate = 1.2 mL·h^−1^. (**a**) depending on observation position (Coefficients of determination are $R^{2}=0.95$ for 10 mm, $R^{2}=0.98$ for 35 mm and $R^{2}=0.94$ for 60 mm), (**b**) when detection time is set to 20 min. (*C* = 0.0395, *D* = $2.4\times 10^{-12}\;m^{2}\cdot s^{-1}$, *W*_1_ = 1362 µm, *W*_2_ = 1021 µm are used for Equation (7)).

Figure 15 shows the results of comparing the estimated values by the model with the experimental results depending on the observation position for number density *n* = $1.93\times 10^{14}\;m^{-3}$ and flow rate = 1.2 mL/h. The comparison was performed to examine the difference in binding efficiency according to the observation position of the channel. The observation position was set to 10 mm, 35 mm, and 60 mm from the inlet to exclude the entrance effect. Figure 15b shows the result of plotting the number of bound particles according to the observation position when fixing the detection time to 20 min. As in the experimental results, the number of bound particles exponentially decreased as the detection position moved further away from the inlet.

**Figure 16:**
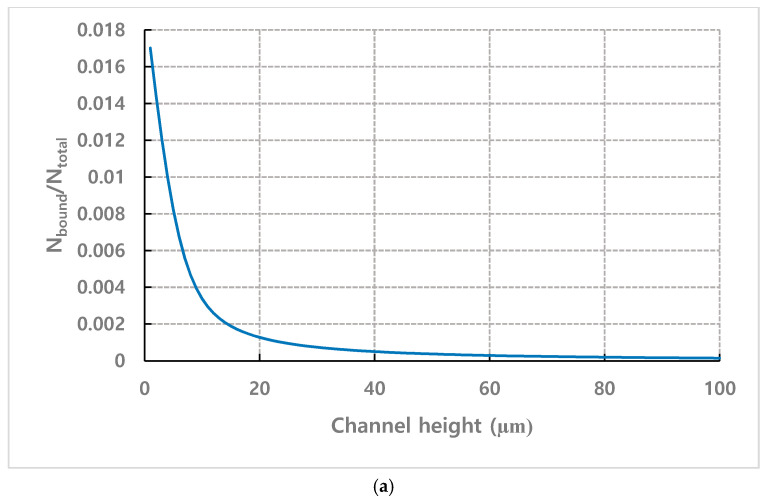

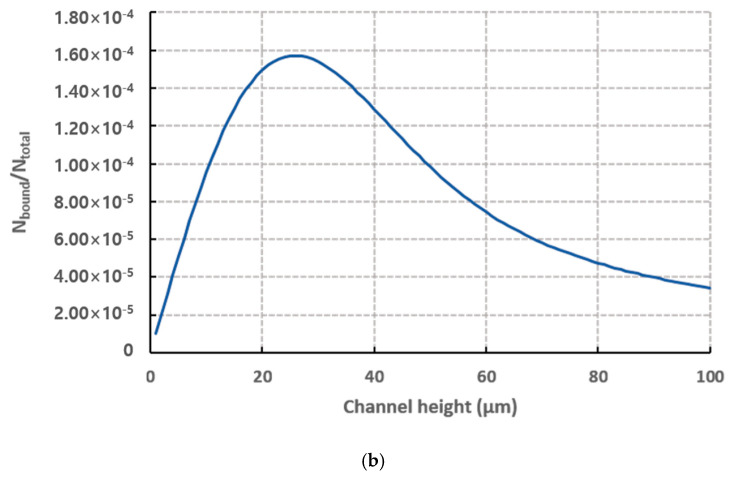
*N*_bound_/*N*_total_ according to the channel height for (**a**) observation position = 0 mm (**b**) observation position = 35 mm.

On the other hand, Figure 16 shows the results of calculating the binding efficiency according to the channel height when fixing the detection time to 20 min and setting the average *x*-direction velocity ($\bar{v_{x}}$) to 0.67 mm·s^−1^, which corresponds to the value at flow rate 1.2 mL·h^−1^ for 100 µm height channel. In this calculation, the binding efficiency is calculated by normalizing the number of bound particles by the total number of injected particles. As shown in Figure 16a, when observing at the inlet, the binding efficiency increases as the channel height decreases. This is because the analytes need to move less laterally to contact receptors on the wall as the height of channel decreases while the mobility by Brownian motion remains the same, regardless of the channel height. On the other hand, when observing at the position 35 mm away from the inlet, the peak for binding efficiency is shifted as shown in Figure 16b. As the channel height decreases, the particles flow away further from the wall may be bound to the receptor on wall, resulting in less particles passing to downstream. For higher channel, only particles closer to the wall can be bound to the wall near the inlet and more particles passes through the area near inlet to touch the wall away from the inlet since the particles need more time to travel before touching the wall by lateral movement. The optimal channel height for maximum binding efficiency tends to increase as the observation position moves further away from the inlet. Therefore, when setting the observation position to a certain distance from the inlet, the channel should be designed by considering the optimal value of the binding probability according to the observation position.

## 5. Conclusions

This article investigates experimentally and mathematically the process of analyte-receptor binding within the channel for a microfluidic biosensor. A mathematical model is proposed based on the probability density function for Brownian motion. Results from analyte-receptor binding experiments are also analyzed and a key coefficient is estimated empirically for the proposed mathematical model. The flow rate, observation position and analyte concentration are used as parameters for the binding efficiency of the microfluidic biosensor. The validity of the model is established by comparing the experimental results. Using the proposed mathematical model, an example of micro-fluidic channel for biosensor is designed and investigated the effect of the channel dimension on the binding efficiency. This study also presented an index for device design to optimize the binding efficiency, which may be used to enhance the limit of detection or to reduce the detection time or the amount of the samples. 

The viscosity, temperature, interaction between analyte, or electrostatic properties such as Debye length can affect the motion of analytes. In addition, the binding phenomenon can be influenced by the chemical properties such as acidity of the sample solution. In this study, these properties were fixed in order to examine the basic motion of analyte through microfluidic channel. Hence, further research about these properties is needed. 

## Figures and Tables

**Table 1:** Experimental conditions.

Number Density (*n*)	Flow Rate	Observation Position (*L*)
$1.93\times 10^{15}\;m^{-3}$	1.2 mL·h^−1^	35 mm
$1.93\times 10^{14}\;m^{-3}$	1.2 mL·h^−1^	35 mm
$1.93\times 10^{13}\;m^{-3}$	1.2 mL·h^−1^	35 mm
$1.93\times 10^{12}\;m^{-3}$	1.2 mL·h^−1^	35 mm
$1.93\times 10^{14}\;m^{-3}$	0.3 mL·h^−1^	35 mm
$1.93\times 10^{14}\;m^{-3}$	2.4 mL·h^−1^	35 mm
$1.93\times 10^{14}\;m^{-3}$	1.2 mL·h^−1^	10 mm
$1.93\times 10^{14}\;m^{-3}$	1.2 mL·h^−1^	60 mm
